# Genotype-Dependent Interaction of Lentil Lines with *Ascochyta lentis*

**DOI:** 10.3389/fpls.2017.00764

**Published:** 2017-05-10

**Authors:** Ehsan Sari, Vijai Bhadauria, Albert Vandenberg, Sabine Banniza

**Affiliations:** Department of Plant Sciences/Crop Development Centre, University of Saskatchewan, Saskatoon,SK, Canada

**Keywords:** histology, cell death, salicylic acid, jasmonic acid, PR-genes

## Abstract

Ascochyta blight of lentil is a prevalent disease in many lentil producing regions and can cause major yield and grain quality losses. The most environmentally acceptable and economically profitable method of control is to develop varieties with high levels of durable resistance. Genetic studies to date suggest that ascochyta blight resistance genes (R-gene) in lentil lines CDC Robin, ILL 7537, 964a-46, and ILL 1704 are non-allelic. To understand how different R-genes manifest resistance in these genotypes and an accession of *Lens ervoides*, L-01-827A, with high level of resistance to ascochyta blight, cellular and molecular defense responses were compared after inoculation with the causal pathogen *Ascochyta lentis*. Pathogenicity testing of the resistant lines to *A. lentis* inoculation revealed significantly lower disease severity on CDC Robin and ILL 7537 compared to ILL 1704 and 964a-46, and no symptoms of disease were observed on L-01-827A. Histological examinations indicated that cell death triggered by the pathogen might be disrupted as a mechanism of resistance in CDC Robin. In contrast, limiting colonization of epidermal cells by *A. lentis* is a suggested mechanism of resistance in 964a-46. A time-series comparison of the expressions of hallmark genes in salicylic acid (SA) and jasmonic acid (JA) signal transduction pathways between CDC Robin and 964a-46 was conducted. These partially resistant genotypes differed in the timing and the magnitude of SA and JA signaling pathway activation. The SA signaling pathway was only triggered in 964a-46, whereas the JA pathway was triggered in both partially resistant genotypes CDC Robin and 964a-46. The expression of JA-associated genes was lower in 964a-46 than CDC Robin. These observations corroborate the existence of diverse ascochyta blight resistance mechanisms in lentil genotypes carrying different R-genes.

## Introduction

Ascochyta blight of lentil (*Lens culinaris* Medik.) caused by *Ascochyta lentis* Vassilievsky (teleomorph: *Didymella lentis* W.J. Kaiser, B.C. Wang, and J.D. Rogers) is prevalent throughout many temperate lentil production regions of the world and has been reported to cause yield losses of up to 70% in Canada, 30–50% in the USA, and 50% in Australia (Gossen and Morrall, 1983; Kaiser, 1992; Brouwer et al., 1995). The most environmentally acceptable and economically profitable method of control is to develop varieties with high levels of durable resistance. A few major ascochyta blight R-genes have been characterized in different lentil genotypes (Tay and Slinkard, 1989; Andrahennadi, 1994, 1997; Ahmad et al., 1997; Ford et al., 1999; Ye et al., 2000; Nguyen et al., 2001), and varieties partially resistant to ascochyta blight have been released (Ali, 1995; Vandenberg et al., 2001, 2002).

Due to continuous exposure to insects and pathogens, plants are armed with a sophisticated immune system that recognizes various types of stimuli and responds accordingly by activating intricate and effective defense pathways (Jones and Dangl, 2006; Howe and Jander, 2008). Conclusive evidence points to the involvement of the phytohormones SA, JA, ethylene (ET), and abscisic acid (ABA) as primary signals in fine-tuning the plant immune system (Pieterse et al., 2009; Verhage et al., 2010). The accumulation of individual or blends of phytohormones upon pathogen challenge can generally be linked to the infection strategy of pathogens. The SA-dependent pathway induces resistance against biotrophic pathogens, but is also activated upon invasion by hemi-biotrophs. The JA/ET induces resistance against necrotrophs and hemibiotrophs (Kunkel and Brooks, 2002). The defense responses induced by the ABA signaling pathway are more complicated, and both, augmented resistance and susceptibility to pathogens have been reported in ABA defective mutants (Ton et al., 2009). By balancing the biosynthesis of these signaling compounds through an intricate network of cross-talk, plants are able to spatially and temporarily adjust their defense responses (Pieterse et al., 2009). However, compatible pathogens can harness these pathways to their own benefit by secreting effectors that directly or indirectly antagonize the host immune responses (Pieterse and Dicke, 2007; Grant and Jones, 2009). Recent evidence suggests that some necrotrophs even hijack resistance mechanisms that are effective against biotrophs to induce cell death and promote host cell colonization (Hammond-Kosack and Rudd, 2008; Kazan and Lyons, 2014).

As separate groups of pathogenesis-related (PR) proteins are induced when SA and JA/ET pathways are triggered, pathway-specific PR proteins have frequently been used to indirectly monitor the activation of SA and JA/ET signaling in various plant-pathogen interaction studies (e.g., Penninckx et al., 1996; Lorenzo et al., 2003). Previous studies revealed the requirement of SA signaling for induction of *PR-1*, *PR-2*, and *PR-5*, and JA signaling for *plant defensin like protein 1.2* (*PDF1.2)*, *hevein-like protein* (*HEL*), *basic chitinase* (*CHI-B*), *PR-3*, and *PR-4* (Thomma et al., 1998). *PR-1* has been widely accepted as a hallmark of SA signaling in *Arabidopsis thaliana* (L.) Heynh. (Rogers and Ausubel, 1997) and some crop species such as tomato (*Lycopersicon esculentum* Mill., Niderman et al., 1995; Tornero et al., 1997). PR-1 proteins also appear to possess anti-microbial activity (Alexander et al., 1993). Proteins of the PR-5 family are homologous to thaumatin- and osmotin-like proteins and show destructive effects on the permeability of fungal plasma membranes (Abad et al., 1996). The only PR proteins studied in lentil to date are those of the PR-4 family. Transcriptome analysis of lentil genotypes partially resistant to ascochyta blight revealed up-regulation of *PR-4* upon pathogen challenge in the partially resistant but not in the susceptible genotype (Mustafa et al., 2009). The antifungal activity of PR-4 proteins has been shown in other plant-pathogen systems (Caruso et al., 2001). Vaghefi et al. (2013) demonstrated *in vitro* the antifungal activity of a recombinant lentil PR-4 protein (LcPR4a) on *A. lentis.*

Allene oxidase cyclase is a key enzyme in the JA pathway, involved in JA biosynthesis from α-linolenic acid (Vick and Zimmerman, 1983). The *AOC* gene has been cloned from plants such as *Arabidopsis* (Stenzel et al., 2003), *Lycopersicon* (Ziegler et al., 2000), and *Medicago truncatula* Gaertn and is of primary importance in JA signaling for legume mycorrhization (Isayenkov et al., 2005). Moreover, *AOC* has potential utility as a marker for monitoring the JA signaling pathway (Leon-Reyes et al., 2010).

Microscopic examination of cellular reactions to a plant pathogen have been widely used in the study of plant-fungal interactions (Hood and Shew, 1996). Success in microscopic studies depends on the application of staining techniques that allow differentiation of plant and pathogen tissues, enabling detection of cascades of cytological events after infection. Understanding the developmental stages of a pathogen in time and space in the host plant is a prerequisite for determining the sampling intervals required for gene expression analysis of plant-pathogen interactions, and allows gene expression profiles to be correlated with cellular events. The initial infection process of *A. lentis* was studied by Roundhill et al. (1995), who showed that colonization of epidermal cells by *A. lentis* occurred after the disruption of cytoplasm indicating that *A. lentis* is either a necrotroph or hemi-biotroph with a short biotrophic phase. Recently, Sambasivam et al. (2017) compared the cellular reaction of lentil genotypes to two isolates of *A. lentis* with distinctly different levels of virulence. They found that the resistant genotypes reacted faster to pathogen infection, resulting in delayed and reduced formation of the fungal infection structures. The rapid generation of H_2_O_2_ and triggering of the hypersensitive reaction was reported as a common early response to fungal penetration in the resistant genotypes.

Research on fusarium headblight resistant wheat (Foroud et al., 2012) and powdery mildew resistant tomato lines (Bai et al., 2005) has shown that different R-genes can confer resistance through different mechanisms. The non-allelic nature of several ascochyta blight R-genes widely used in lentil breeding programs for the development of partially resistant varieties was recently confirmed (Sari, 2014). Based on the hypothesis that these non-allelic R-genes trigger different resistance mechanisms, the present study was designed to determine whether lentil genotypes carrying non-allelic R-genes differ in their cellular reactions to *A. lentis* infection. For two genotypes, the differential activation of SA and JA signal transduction pathways was also assessed.

## Materials and Methods

### Plant Materials

Four lentil genotypes that have been widely used for improving resistance to ascochyta blight in lentil breeding programs were used in this study: CDC Robin, 964a-46, ILL 1704, and ILL 7537. Also included were *L. ervoides* (Brign.) Grande accession L-01-827A (a single plant selection from the ICARDA accession IG 72847, Fiala et al., 2009) and lentil cv. Eston (susceptible control). CDC Robin has a recessive ascochyta blight resistance R-gene (*ral2*) derived from cultivar Indianhead and is also partially resistant to race 1 of *Colletotrichum lentis* Damm causing anthracnose (Andrahennadi, 1994; Vail et al., 2012). Breeding line 964a-46 has a dominant R-gene (*AbR1*) derived from ILL 5588, which is also the source of resistance for cv. Northfield (Ali, 1995). CDC Robin, 964a-46, and Eston were developed at the Crop Development Centre (CDC), University of Saskatchewan, Canada (Slinkard, 1981; Vandenberg et al., 2002). ILL 7537 and ILL 1704 are landraces from Jordan and Ethiopia, respectively, with resistance to ascochyta blight as reported in previous studies (Rubeena et al., 2006; Tullu et al., 2010). Nguyen et al. (2001) showed that ILL 7537 carries a R-gene different from that in ILL 5588. Using recombinant inbred lines developed from crosses among four partially resistance lines, Sari (2014) determined that non-allelic R-genes condition resistance to ascochyta blight in ILL 7537, CDC Robin, 964a-46, and ILL 1704. Analysis of a population derived from Eston and *L. ervoides* L-01-827A indicated the presence of two complementary recessive ascochyta blight R-genes in L-01-827A (Sari, 2014).

### Inoculation Procedure and Ascochyta Blight Disease Severity Rating

A conidial suspension was prepared from a monoconidial culture of *A. lentis* isolate AL57, an aggressive isolate from Landis, Saskatchewan, Canada (Banniza and Vandenberg, 2006). AL57 was stored in a cryopreservation solution containing 10% skim milk and 20% glycerol at -80°C. Spores were revitalized on 50% oatmeal agar plates (30 g oatmeal [Quick Oats, Quaker Oats Co., Chicago, IL, USA], 8.8 g agar [Difco, BD^^®^^, Sparks Glencoe, MD, USA], 1 L H_2_O), and incubated for 7 days at room temperature. The spore suspension was prepared following the protocol described by Vail and Banniza (2008). The concentration of the spore suspension was adjusted to 5 × 10^5^ conidia mL^-1^ using a hemocytometer.

Four seeds of each lentil genotype were sown in 10 cm square pots containing a mixture of Sunshine Mix No. 4 (Sun Grow Horticulture^^®^^ Ltd., Vancouver, BC, Canada) and Perlite^TM^ (3/1 V/V). Pots were maintained for 21 days in a greenhouse with average daily temperature of 23.5°C, relative humidity of 66% and a 18 h/6 h day/night light regime supplied from the integration of natural an artificial lighting. Seedlings with 10–15 expanded leaves were inoculated with the spore suspension at a rate of 2 mL per seedling using an airbrush, and were incubated in a humidity chamber for 48 h. Since L-01-827A grew slower than the other lentil genotypes, inoculation was repeated 2 weeks after the first one for this genotype to rule out any confounding effect of growth stage with host response in this genotype. Plants were then incubated under the same greenhouse conditions as before, but on a mist bench where they were misted with water for 30 s every 90 min during the day for the remainder of the test. The experiment was conducted as a randomized complete block design with four replicates and was conducted twice. Disease severity data were collected for each of the four plants grown in each pot, 3 weeks after inoculation, using a scale of 1–10 based on 10% incremental increases in the percentage of symptomatic area on leaflets and stems. Data were converted to percentage disease severity using the class midpoints and averaged across the four plants for each replicate for data analysis.

### Microscopy of Cellular Reaction of Lentil Genotypes to *A. lentis* Infection

#### Quantitative Assessment of Fungal Infection Structures

Quantitative microscopy was used to investigate how defense mechanisms counteracted the growth and development of *A. lentis* on each lentil genotype. The experiment was conducted as a randomized complete block design with three replicates and was conducted twice. Plants were grown and inoculated as described for pathogenicity testing. All inoculated leaflets of four lentil seedlings grown in one pot were pooled, representing one biological replicate, after collection at 10, 12, 24, 30, and 48 hpi). Fungal structures were stained with Uvitex-2b (Polyscience Inc., Warrington, PA, USA) following the protocol of Moldenhauer et al. (2006) with minor modification. Leaflet tissue was immersed in ethanol-chloroform (3:1, v/v) containing 0.15% (w/v) trichloroacetic acid immediately after collection and cleared for at least 18 h followed by washing in 50% ethanol. Leaflets were then soaked in 0.1 M Tris-HCL buffer (pH = 5.8) for 30 min and stained in 0.1% (w/v) Uvitex-2b in 0.1 M Tris-HCL buffer (pH = 5.8) for 5 min. Samples were de-stained by washing four times for 10 min in water. Specimens were mounted in 50% glycerol for slide preparation.

Three leaflets were arbitrarily selected from the pool of leaflets for each biological replicate of each treatment and subjected to quantitative measurements. Percentage of conidial germination was determined for samples collected at 10 and 12 hpi by examining 100 conidia per three fields of vision. Conidia were considered germinated when they produced germ tubes equal to or longer than the conidial diameter. To determine the length of infectious hyphae, leaflets collected at 24, 30, and 48 hpi were examined in 10 fields of vision (each containing a minimum of 10 germinated conidia) and images were recorded for each field using an AxioCamICc1 digital camera installed on a Zeiss Axioplan fluorescent microscope (Carl Zeiss, Göttingen, Germany). The length of infectious hyphae was determined using the curve spline tool of Axiovision 4.7 digital image processing software. All quantitative data were collected with BP excitation/emission cubes (546/FT580/LP590).

#### Description of Epidermal Cell Response to *A. lentis* Infection Using Confocal Laser Scanning Microscopy

For CDC Robin, 964a-46 and Eston descriptive microscopy was used to determine the underlying cellular mechanisms of defense and differences among genotypes in cellular reactions to *A. lentis* infection. Ten infected leaflets were arbitrarily selected from the pool of leaflets collected from single plants of each genotype at 60 and 90 hpi, and were discolored and stained with Uvitex-2b following the protocol described above. The reaction of epidermal cells to pathogen penetration was studied using a two photon Carl Zeiss confocal laser scanning microscope as described by Moldenhauer et al. (2006). The specimens were excited with UV-laser beams at 351 and 364 nm, then scanned with filter settings at 400–500 nm for Uvitex 2b-stained fungal structures, and with argon-laser beams at 514 and 543 nm, and then scanned with filter settings at 560–680 nm for epidermal cells responses. Observations of pathogen and plant cells located at different tissue depth were conducted by collecting images in a number of Z stacks at 0.5 μm intervals. The Z stacks were then compiled to a single micrograph using the Z projection tool in Image J 1.7 p (Rasband, W.S., ImageJ, U.S. National Institutes of Health, Bethesda, MD, USA^1^, 1997–2012).

#### Test of Cell Viability by Light Microscopy

The viability of epidermal cells of CDC Robin, 964a-46, and Eston was investigated following the method of O’Connell et al. (1991) with the following modifications. Samples of 10 infected leaflets, arbitrarily selected from the pool of leaflets collected from single plants of each genotype at 60 and 72 hpi, were subjected to viability staining as follows: Leaflets were cut in half and then vacuum-infiltrated in 0.85 M KNO_3_ containing 0.01% Neutral Red (Sigma-Aldrich, St. Louis, MO, USA) for 5 h. The specimens were then mounted in the infiltration solution and fungal structures stained with a drop of 0.1% Aniline Blue (BDH Prolabo, UK) in lactic acid solution. The specimens were examined under a Zeiss light microscope (Carl Zeiss, Göttingen, Germany) and images were recorded using an AxioCamICc1 digital camera.

### Analysis of the SA and JA Signal Transduction Pathways by qRT-PCR

The temporal pattern of SA and JA signaling after *A. lentis* infection was indirectly assessed by expression analysis of *PR-1* and *PR-5* as hallmarks of the SA pathway, and *PR-4* and *AOC* as hallmarks of the JA pathway. Lentil genotypes CDC Robin and 964a-46 were selected for this test, with the addition of Eston as the susceptible control. Plants were inoculated as described for pathogenicity testing, except that a higher concentration of conidia (10^6^ conidia mL^-1^) was used. The experiment was arranged in a randomized complete block design with three replicates.

All inoculated leaflets of seedlings were collected at 6, 12, 18, 24, 36, 48, and 60 hpi and flash frozen in liquid nitrogen. Leaflets were also collected from non-inoculated control plants sprayed only with water. Leaflet samples were stored at -80°C. Leaflets pooled for each biological replicate were ground in an RNAse-free mortar, pre-cooled with liquid nitrogen. Two subsamples from the pool of ground tissue collected for each biological replicate were subjected to RNA extraction. RNA was extracted using Trizol^^®^^ reagent (Invitrogen, Carlsbad, CA, USA) following the manufacturer’s instructions. Total RNA was then treated with DNAse I (Invitrogen, Carlsbad, CA, USA) to remove any trace of genomic DNA contamination according to the manufacturer’s recommendation. The purity and quantity of RNA were determined using a NanoDrop ND8000 (Thermo Scientific, Wilmington, DE, USA). Samples with an A260/280 ratio less than 2.0 were discarded. The integrity of RNA was determined by denaturing agarose gel electrophoresis (Barill and Nates, 2012).

Total RNA (1 μg) was used for reverse transcriptase-dependent first strand cDNA synthesis, primed by Oligo(dt)_12-18_ primer (Invitrogen, Carlsbad, CA, USA) according to Klickstein et al. (2001). Residual genomic DNA contamination of total RNA samples was detected by running a PCR using ubiquitous actin primer pairs designed for an exon-exon junction and first strand cDNA following the protocol of Vaghefi et al. (2013). PCR was conducted in a 20 μL reaction mix containing 4 μL of 1:10 diluted cDNA, 1X taq reaction buffer, 0.13 μM of each primer, 0.25 mM dNTPs, 3 mM MgCl_2_, and 1 U Taq polymerase (GenScript, Piscataway, NJ, USA). The PCR cycles were 3 min at 95°C, followed by 40 cycles of 30 s at 95°C, 30 s at 57°C, and 30 s at 72°C, followed by a final extension of 72°C for 7 min. PCR products were visualized by staining with 1:1000 dilution of GelRed^^®^^ (Invitrogen, Carlsbad, CA, USA) added to the loading dye, after separation on a 1.4% agarose gel. Samples with genomic DNA contamination were discarded and cDNA synthesis was repeated after total RNA treatment with a doubled concentration of DNAse I.

Primer sequences of Vaghefi et al. (2013) were used for *PR-4*, whereas primer pairs for *PR-1* and *PR-5* (LcPR-1 and LcPR-5) were designed using the mRNA sequences available in the Expressed Sequence Tag (EST) library of lentil infected with the *C. lentis* (Bhadauria et al., 2013). The mRNA sequence of *β-actin* and *AOC* of *M. truncatula* were used to retrieve their orthologs in lentil cv. Redberry transcriptome using the BLASTn tool available at http://knowpulse.usask.ca/portal/blast/nucleotide/nucleotide (**Table 1**). LcActin-257 and LcAOC-69 were selected from a group of primers designed for lentil *β-actin* and *AOC*, respectively, based on their high fidelity and amplification efficiency. Primers were designed using the primer BLAST search tool provided by the National Centre for Biotechnology Information (NCBI^2^).

**Table 1:** Names, sequences, and gene bank accession number of source sequences of gene-specific primer pairs used for quantitative real-time PCR.

Primer name	Sequence 5′→3′	GenBank accession
LcPR-1	F: AGATCCGAGGTTGGTGTTTC	JG294109
	R: CCCACAATTTCACAGCATCT	
LcPR-5	F: CACTGTATGGCCAGGAACAC	JG293995
	R: TACCAAAGTTGCTGGTGGAA	
LcAOC-69	F: AGAGTAGGCATAACTGCAGGCT	AJ866733^∗^
	R: TGGTACGTCAGATAAGCTCCCTGT	
LcActin-257	F: CACTGTACTTCCTCTCCGGC	EU664318^∗^
	R: TATGTTCCCCGGGATTGCTG	

*^∗^The mRNA sequence of β-actin and Allen Oxidase Cyclase of Medicago truncatula were used to identify their orthologs in lentil cv. Redberry transcriptome using the BLASTn tool available at http://knowpulse.usask.ca/portal/blast/nucleotide/nucleotide.*

The qPCR reaction included 10 μL of Power SYBR^^®^^ Green master mix (Applied Biosystems, Warrington, UK), 0.2 μM of each primer, and 5 μL of 1:10 diluted cDNA. The cycling program was executed in an ABI StepOnePlus^TM^ Real-Time PCR machine (Applied Biosystems, Foster City, CA, USA) and comprised 95°C for 10 min, 40 cycles of 95°C for 30 s, 60°C for 1 min, and 72°C for 30 s followed by a melting curve from 60 to 95°C with 0.3°C intervals. PCR was conducted in duplicate. The expression level was reported relative to the non-inoculated control by calculating fold changes following the method of Livak and Schmittgen (2001).

Amplification efficiency was calculated for each primer pair using cDNA samples serially diluted 1:4 (v/v) five times (total six dilutions). Dilutions were used as a template for qPCR following the protocol described above. A linear equation was fitted to the cycle threshold (*C*_t_) values obtained for various cDNA dilutions. Percentile of amplification efficiency (*E*) was calculated from the slope of the regression line using the equation *E* = 10^(-1/slope)^-1.

### Statistical Analysis

All data analyses were performed using Statistical Analysis System (SAS) version 9.3 (SAS Institute Inc., Cary, NC, USA). Homogeneity of variances was tested using the Levene’s test and, in the case of heterogeneity, the variances were modeled using the SAS mixed model procedure.

Percentage of disease severity data were subjected to mixed model analysis with genotypes assigned as fixed, block nested in repeat and repeat as random effects. Quantitative microscopy data were subjected to mixed model analysis with genotypes and sampling time points assigned as fixed, block nested in repeat and repeat as random and sampling time points as repeated measure effects. Means of conidial germination and length of infectious hyphae on the genotypes were compared at each individual time point based on least significant differences with the Tukey adjustment (α = 0.05).

For statistical analysis of qRT-PCR data, the mean *C*_t_ of two technical qPCR replicates was normalized (Δ*C*_t_) and converted to 2^-Δ^*^C^*^t^. Data were subjected to generalized linear mixed model analysis using the SAS generalized linear mixed model procedure. Genotypes and sampling time points were considered fixed effects, replicates were random effects and sampling time points were identified as repeated measurements. A log-normal distribution with an identity link function was specified to account for the non-normal distribution, and a first-order ante-dependence covariance structure was used to accommodate unequally spaced sampling time points and heterogeneous variances. Differences among genotypes and sampling time points were assessed based on least significant differences with the Tukey adjustment (α = 0.05) in the generalized linear mixed model procedure. To confirm the validity of the reference gene (*β-actin*) for normalization, the *C*_t_ values generated for LcActin-257 primer of non-inoculated leaflets were compared with those of inoculated samples using the Kruskal–Wallis test (Schmittgen and Zakrajsek, 2000).

## Results

### Reaction of *Lens* Genotypes to *A. lentis* Inoculation

**FIGURE 1:**
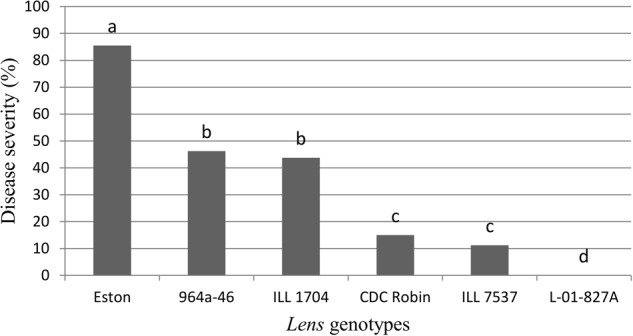
**Percentage of ascochyta blight severity of *Lens* genotypes.** All genotypes are accession of *L. culinaris* except L-01-827A (*Lens ervoides*). Estimates of the means were generated from four biological replicates using a mixed model analysis. Disease severity was rated using a 1–10 scale with 10% incremental increase in disease severity and converted to percentage disease severity using the class midpoint. Means with one letter in common are not significantly different.

Genotypes had a significant effect on disease severity (*P* = 0.0263), and all the partially resistant genotypes had lower disease severity than the susceptible control Eston (**Figure 1**). No symptoms were observed on *L. ervoides* L-01-827A despite a second inoculation 2 weeks after the first one, indicating that a high level of resistance to ascochyta blight is age-independent. Disease severity was not different between CDC Robin and ILL 7537, but both had significantly lower disease severity than the partially resistant genotypes ILL 1704 and 964a-46. There was no significant difference in ascochyta blight severity between ILL 1704 and 964a-46.

### Quantitative Assessment of Fungal Infection Structures

**FIGURE 2:**
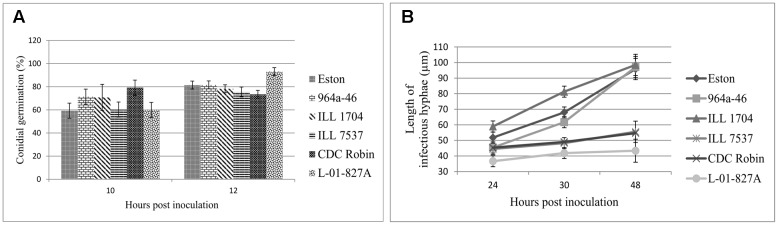
**Mean germination (%) of conidia (A)**, and mean length of infectious hyphae (μm; **B**) extending from germinated *Ascochyta lentis* conidia on the leaflet surface of *Lens* genotypes at 10 to 48 hpi). All genotypes are *L. culinaris* except L-01-827A (*L. ervoides*). Estimates of means were generated from three biological replicates using a mixed model analysis. For each biological replicate 300 conidia were assessed for conidial germination, and a minimum of 100 for infectious hyphae length.

Percent conidial germination was determined for lentil genotypes to investigate the potential association between germination inhibition and resistance to ascochyta blight. Conidial germination was a host genotype-independent trait as differences among genotypes were not significant (*P* = 0.47). Incubation time (*P* = 0.0012) and the interaction (*P* = 0.04) had significant effects on germination, and germination significantly increased from 10 to 12 hpi for Eston and L-01-827A, but not for the other genotypes. Percent conidia germination at 12 hpi ranged from 73.4 to 93% and *L. ervoides* accession L-01-827A and CDC Robin had the highest and lowest germination rate, respectively (**Figure 2A**).

As a second step to understanding the point at which *A. lentis* growth was inhibited in the resistant genotypes, the length of infectious hyphae was measured at three time points to cover post-penetration stages of infection. Analysis of variance showed significant effects of genotypes (*P* < 0.001), sampling time points (*P* < 0.001), and their interaction (*P* = 0.0019), suggesting that length of infectious hyphae was a genotype-dependent trait and the effect of genotypes on the length of infectious hyphae changed over time. For all genotypes, the length of infectious hyphae increased over time (**Figure 2B**). At 24 hpi, conidia on ILL 1704 had developed longer infectious hyphae than on all other genotypes except for Eston. Increasing incubation time to 30 and 48 h resulted in consistently shorter infectious hyphae in CDC Robin, ILL 7537, and L-01-827A compared to ILL 1704, 964a-46 and Eston. No significant differences in the length of infectious hyphae were observed between 964a-46 and Eston at any time point. Based on the length of infectious hyphae, lentil genotypes could be separated into two groups, one with restricted infectious hyphae growth (CDC Robin, ILL 7537 and L-01-827A), and one with long infectious hyphae (ILL 1704, 964a-46 and Eston).

### Cellular Reaction of Lentil Genotypes to Infection by *A. lentis*

Partially resistant genotypes CDC Robin and 964a-46 were selected for descriptive microscopy as representatives of the two groups with short and long infectious hyphae, respectively, and cellular reactions were compared with the susceptible genotype Eston.

**FIGURE 3:**
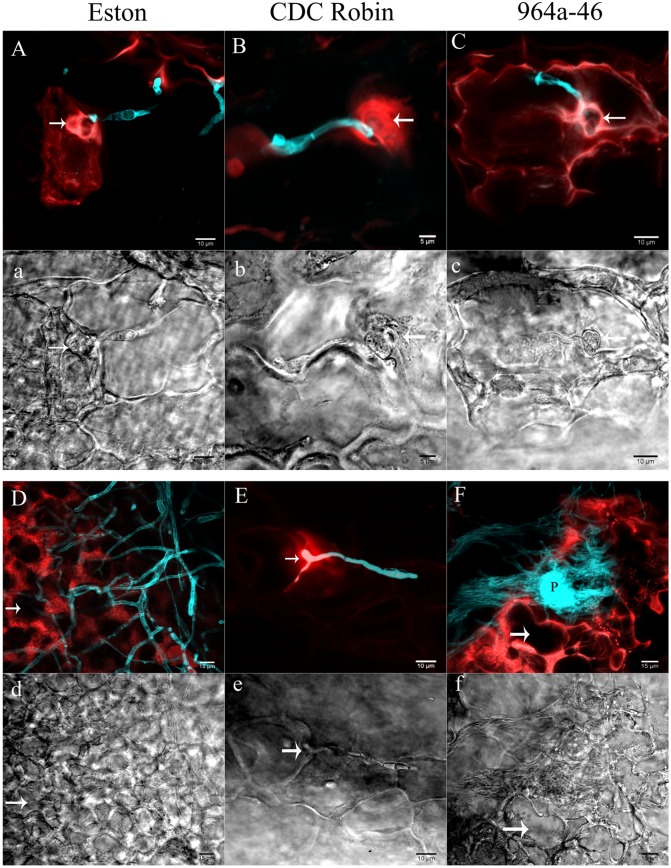
**Cellular reactions of partially resistant lentil genotypes to *A. lentis* infection captured by two photon CLSM at 60 (A,a,B,b,C,c)** and 90 hpi **(D,d,E,e,F,f)**. Microscopic fields of vision were simultaneously scanned using fluorescence (capital letters) and differential interference contrast (DIC) filters (small letters). Fungal structures (cyan) were stained with Uvitex-2b. Autofluorescent signals developed in the host epidermal cells in response to pathogen infection are in red. Arrows in images show the penetration site except for **(d)** and **(f)**, where arrows indicate the cavity developed as a result of the destruction of cell contents. P in **(F)** shows a newly developed pycnidium formed on the mass of mycelium. Scale bars are indicated at the bottom right of each image.

In Eston, the fluorescent signals emitted from the entire cell protoplast in response to infection. Cell wall reinforcement and papillae at the site of penetration attempts was observed in Eston at 60 hpi (indicated by arrow in **Figures 3A,a**). Cellular events induced following *A. lentis* infection in 964a-46 were similar to those of Eston at 60 hpi (**Figures 3C,c**), although 964a-46 developed thinner papillae compared to Eston. Destruction of papillae by infection vesicles was detected in both 964a-46 and Eston at 60 hpi. In CDC Robin, a concentrated autofluorescent signal was detected at the site of penetration attempts at 60 hpi, and the destruction of papillae was not observed in this genotype at this time point (**Figures 3B,b**).

The reactions of CDC Robin epidermal cells to *A. lentis* did not change from 60 to 90 hpi (**Figures 3B,b,E,e**), unlike those of Eston and 964a-46 where massive colonization of epidermal cells was observed at 90 hpi (**Figures 3D,d,F,f**). The colonization by fungal mycelium was denser in 964a-46 compared to Eston and pycnidia were often developed by 90 hpi in 964a-46.

**FIGURE 4:**
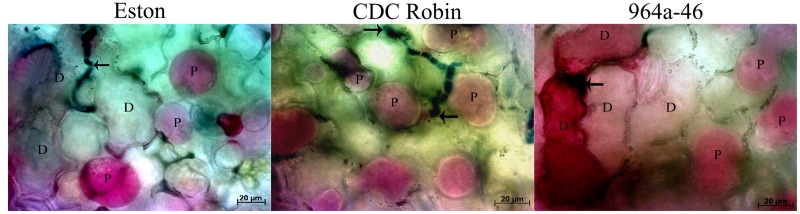
**Changes in viability of epidermal lentil cells 72 h after inoculation with *A. lentis.*** Fungal tissues were stained with Aniline Blue-lactic acid solution (dark blue). Arrows indicate the penetration site. Viability was postulated when the host protoplasm (P) contracted and absorbed red pigments after vacuum infiltration of leaflet tissues in 0.85 M KNO_3_/0.01% Neutral Red solution. Dead cells (D) absorbed the red pigments but did not show contracted protoplasm. Scale bars are indicated at the bottom right of each image.

Results of cell viability test showed that cell death occurred in Eston and 964a-46 at 72 hpi, but not in CDC Robin (**Figure 4**). In Eston, most cells attacked by the pathogen had lost their viability. Cell death was detected in a few non-infected cells neighboring the infection site in 964a-46 at 72 hpi. Penetration into epidermal cells was observed in Eston and 964a-46 but not in CDC Robin at this time point.

### Quantitative Measurement of Gene Expression of *PR-1*, *PR-5*, *PR-4*, and *AOC*

All gene-specific primers had amplification efficiencies close to 100% (data not presented). No significant differences were observed between the *C*_t_ values generated for the LcActin-257 primer of non-inoculated leaflets and those of inoculated samples (Eston: *P* = 0.4414; CDC Robin: *P* = 0.4159; 964a-46: *P* = 0.1037). Variance analyses showed that genotype (*P* < 0.0001) and incubation time (*P* < 0.0001) and their interaction had very highly significant effects on the expression of *PR-1*, *PR-4*, *PR-5*, and *AOC.*

**FIGURE 5:**
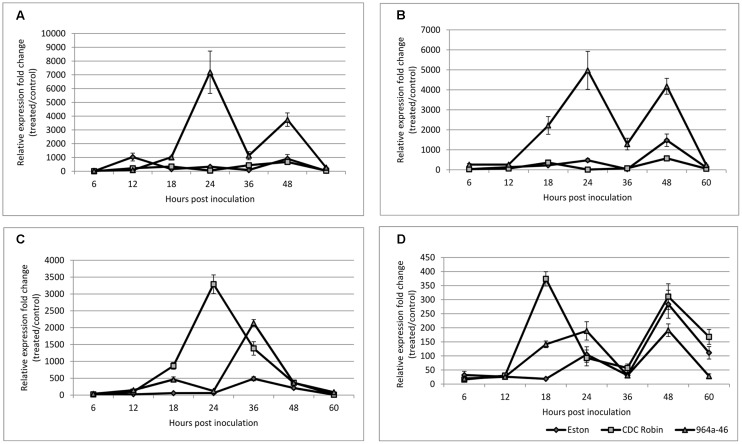
**Quantitative assessment of *PR-1* (A)**, *PR-5* **(B)**, *PR-4* **(C)**, and *AOC* **(D)** expression in susceptible lentil genotype Eston and two partially resistant CDC Robin and 964a-46 by quantitative real-time PCR after inoculation with *A. lentis.* Data are mean of three replicates. Error bars indicate standard errors of the mean. Gene expression was reported relative to non-inoculated samples collected just prior to inoculation. Data were normalized using *β-actin* gene expression as a reference gene.

CDC Robin and Eston were similar in relative *PR-1* expression over the tested time points except for 12 hpi, when *PR-1* expression in Eston was significantly higher than in CDC Robin (**Figure 5A**). *PR-1* expression in 964a-46 increased exponentially at 18 hpi and peaked at 24 hpi, when expression was estimated to be 7084 times higher than in non-inoculated samples. The expression then declined and all three genotypes had similar levels of expression at 36 hpi. Subsequently, the expression increased again in 964a-46, but less rapidly and to a lower peak than the first fold increase starting at 18 hpi.

*PR-5* expression was not significantly different between the susceptible check Eston and CDC Robin at all sampling time points except for 48 hpi (**Figure 5B**). At 48 hpi, Eston had significantly higher *PR-5* expression than CDC Robin, but lower than 964a-46. In 964a-46, *PR-5* expression was not different from the others at 6 and 12 hpi. However, its expression exponentially increased after 12 hpi and reached a peak that was 4910 times that of the non-inoculated plants at 24 hpi. The peak detected in 964a-46 at 24 hpi was negligible in Eston and absent in CDC Robin. Although expression in 964a-46 had declined at 36 hpi, levels were still higher than for CDC Robin and Eston. *PR-5* expression increased again at 48 hpi in 964a-46 to a level similar to that at 24 hpi, then declined at 60 hpi to a level similar to that at 6 and 12 hpi. Eston and CDC Robin experienced an increase in *PR-5* expression at 48 hpi similar to that noted for, but of lower magnitude than in 964a-46. As in 964a-46, *PR-5* expression then declined in CDC Robin and Eston to levels initially observed at 6 and 12 hpi.

*PR-4* expression was the same in all genotypes at 6 and 12 hpi (**Figure 5C**). In Eston, it remained low until 24 hpi, and then increased to a peak at 36 hpi before gradually declining. For CDC Robin, *PR-4* expression increased at 18 hpi, reached a peak level at 24 hpi (3281 times that of non-inoculated plants), and then declined. There were significant differences between the expression levels of *PR-4* in CDC Robin and Eston at 18–36 hpi. *PR-4* expression in 964a-46 increased starting at 18 hpi but began to decline at 24 hpi. At 24 hpi, Eston and 964a-46 had almost identical levels of *PR-4* expression. The expression of *PR-4* increased again for 964a-46 at 36 hpi to levels significantly higher than that observed for CDC Robin and Eston, but then returned to similarly low levels.

Allene oxidase cyclase expression in Eston remained at the baseline until 18 hpi, but increased at this time point for both CDC Robin and 964a-46 (**Figure 5D**). This increase was more than double in CDC Robin compared to 964a-46. *AOC* expression then declined at 24 hpi in CDC Robin, but the decline was not observed for 964a-46, which had significantly higher *AOC* expression at 24 hpi compared to 18 hpi. All genotypes showed a decline in *AOC* expression at 36 hpi, and no significant differences were observed among them. All genotypes experienced a fold-change increase at 48 hpi and a decline in expression at 60 hpi.

## Discussion

This study investigated for the first time the differences in cellular reactions and the activation of SA and JA signaling pathways among lentil genotypes with partial resistance to ascochyta blight in response to *A. lentis* infection. Microscopic examination of infected leaflets of these genotypes indicates that the process of cell death is of relative importance in resistance of lentil to ascochyta blight. Genotypes showed different patterns in the expression of genes connected with the SA and JA signal transduction pathways. The involvement of both the SA and JA pathways in the reaction of lentil to ascochyta blight is implicated. There were differences among genotypes in deployment of the the signaling pathways during the course of infection with *A. lentis*.

Conidial germination was confirmed to be genotype-independent, similar to the results of previous microscopic studies of the related fungal species *A. rabiei* when infecting chickpea (Höhl et al., 1990). Sambasivam et al. (2017) found higher conidial germination for a highly virulent *A. lentis* isolate on three lentil genotypes with different levels of resistance than for a less virulent isolate at 2 hpi, but this difference only persisted on the most resistant genotype ILL7537, indicating that there may be an interaction between isolates and lentil genotypes in terms of germination. After germination, *A. lentis* conidia develop germ tubes that penetrated into the epidermal cells by differentiating to appressoria and penetration pegs. Minor differences in the length of infectious hyphae were apparent at 24 hpi, but starting at 30 hpi, the differences became more obvious. Infectious hyphae observed on partially resistant genotypes CDC Robin, ILL 7537 and L-01-827A were consistently shorter than those on the susceptible genotype Eston. Although not analyzed in detail, Sambasivam et al. (2017) also appear to have observed shorter germ tubes on the resistant than the more susceptible genotypes, lending more supports to an inhibition of host colonization as a common mechanism of resistance to *A. lentis* in lentil. At less than 30% disease severity, CDC Robin, ILL 7537 and L-01-827A had high levels of partial resistance to *A. lentis* isolate AL57 infection. In contrast, on ILL 1704 and 964a-46, the length of infectious hyphae was similar to that of conidia on the susceptible control Eston. In the pathogenicity tests, ILL 1704 and 964a-46 were significantly more resistant than Eston, but had higher disease severity than CDC Robin, ILL 7537, and L-01-827A. These results were the first indications for the involvement of different defense mechanisms, or components thereof, in these lentil genotypes for which resistance is conferred by non-allelic R-genes. Differences during the infection phase on lentil genotypes were previously reported from the hemibiotrophic pathogen *C. lentis* (Armstrong-Cho et al., 2012). Similarly, Kema et al. (1996) found differences in the number of epidermal cells colonized among wheat genotypes that varied in their level of resistance to *Mycosphaerella graminicola* (Fückel) Schroeter.

Assessment of cellular responses by CLSM revealed an accumulation of autofluorescent compounds at the sites of penetration attempts in both susceptible and partially resistant genotypes starting at 60 hpi, but the emission of fluorescent signals from cell protoplasts was only observed in Eston and 964a-46. The viability tests indicated that emission of fluorescent signals from cell protoplasts was due to disruptions of cell protoplasm and cell death. This was also implied by a previous report of microscopic studies of *A. lentis* on two lentil genotypes with different levels of resistance to ascochyta blight (Roundhill et al., 1995). They reported differences among susceptible and resistant genotypes at the penetration stage. While susceptible cells became necrotic, followed by growth of the penetration peg into the cell lumen in that study, the penetration peg on the resistant genotypes was surrounded by electron-dense materials and the cells remained viable. These and the present findings suggest that cell death might facilitate the colonization of epidermal cells by the fungus. Necrotrophic and hemi-biotrophic plant pathogens are dependent on cell death for their pathogenesis and cell death promotes colonization of plants by necrotrophs such as *Botrytis cinerea* Pers.: Fr (Govrin and Levine, 2000). Enhanced cell death *Arabidopsis* mutants show comparatively higher susceptibility to necrotrophs (Veronese et al., 2004).

Additional support for the role of cell death in the pathogenicity of *A. lentis* is that cell death was rarely detected in CDC Robin at 60 and 90 hpi. Rare cases of cell death observed in CDC Robin at 90 hpi correspond with the low levels of ascochyta blight symptoms observed on this genotype. Ascochyta blight resistance identified to date is partial, so the inconsistent inhibition of cell death in CDC Robin may contribute to the partial nature of resistance. Induction of cell death was suggested as a defense response in the resistant genotype ILL7537 (Sambasivam et al., 2017). Allelism tests suggested that ascochyta blight R-genes in CDC Robin and ILL 7537 are different (Sari, 2014), lending support to the presence of different resistance mechanisms between these genotypes.

Similar cascades of cellular events were observed in 964a-46 and Eston, with the only differences being that relatively higher numbers of cells surrounding the infection site lost viability, and relatively denser fungal colonies and thinner papillae formed in 964a-46. The engagement of non-infected cells in 964a-46 may be due to a systemic signal transduced to the neighboring cells. The occurrence of systemic signaling was previously suggested as the main difference between two genotypes of wheat with resistance to fusarium head blight (Foroud et al., 2012). Transduction of systemic signals to non-infected cells around the infection site could have primed defense responses and decreased the aggressiveness of the pathogen, thereby limiting the area of colonization in 964a-46. The formation of thicker papillae in Eston than 964a-46 suggest that papillae are not involved in the *A. lentis* resistance of 964a-46. Further research on the frequency and variation in size of papillae would be required to fully resolve their role in resistance. Overall, microscopic studies could not provide conclusive evidence for phenotypic separation of the colonization process of 964a-46 and the susceptible control Eston. Improvement of microscopy techniques may lead to better phenotypic differentiation in future studies.

Analysis of quantitative expression of *PR-1*, *PR-5*, *PR-4*, and *AOC* suggested that genotypes differed with respect to their activation of the SA and JA signaling pathways. The rapid increase in *PR-1* and *PR-5* expression in 964a-46 at 24 hpi suggests the involvement of the SA pathway in the interaction of this genotype with *A. lentis*. The SA-mediated signaling pathway activates defense responses that are only effective against biotrophic and hemi-biotrophic fungi (Kunkel and Brooks, 2002), but is also part of the host response to hemibiotrophic infection (Liu et al., 2007). Roundhill et al. (1995) previously suggested that *A. lentis* is either a necrotrophic or a hemi-biotrophic fungal pathogen, but activation of both pathways as shown here support a hemi-biotrophic lifestyle of this pathogen. In the case of the hemibiotrophic fungal pathogen *Fusarium graminearum* Schwabe of wheat, the SA pathway was triggered very early at 6 hpi and the levels of expression were higher in resistant than in susceptible lines (Ding et al., 2011).

The increased levels of *PR-1* and *PR-5* expression were transient and declined at 36 hpi in 964a-46. It is likely that *A. lentis* AL57 suppresses SA-mediated plant defense in 964a-46 by deploying effectors interfering with the SA signaling pathway. Effectors interfering with the SA pathway have previously been identified in various types of plant pathogens. Recently, Liu et al. (2014) reported the secretion of isochorismatases by *Phytophthora sojae* Kaufm. & Gerd. and *Verticillium dahlia* Kleb. that degrades the SA precursor isochorismate and suppress the defense responses induced by the SA signaling pathway. The decline in SA-related genes at 36 hpi could also be attributed to physiological factor affecting photosynthesis. Seyfferth and Tsuda (2014) indicated that “pathogen-induced SA is mainly synthesized via the isochorismate pathway in chloroplasts.” Plants were incubated in humidity chambers for 48 h after inoculation, where they were exposed to a dark period at 36 hpi. Prevention of photosynthesis at 36 hpi might explain the lower production of SA and thus the decline in the expression of SA-depended genes. Similarly, Mouradov et al. (1994) also found a decline followed by a peek in the accumulation of *PR-1* mRNA in barley leaves challenged with *Erysiphe graminis f.sp. hordei* Marchal. The fact that the transient activation of SA signaling was not observed in Eston and CD Robin suggests that these genotypes may not perceive the pathogen in a similar way as 964a-46, either as a result of the successful manipulation of the defense apparatus by the pathogen or due to lack of the receptor genes, respectively.

In a study of the *A. lentis* transcriptome, the involvement of a complex toxin model was proposed in which the quantitative nature of resistance in lentil is attributed to the interactions of numerous toxins produced by the pathogen with their hypothetical corresponding susceptibility factors in the plant (Lichtenzveig et al., 2012). Notably, reactions to ascochyta blight in 964a-46 varied from highly to moderately resistant when challenged with two different isolates of *A. lentis* (Tar’an et al., 2003). The reactivation of the SA pathway in 964a-46 after 36 hpi may be due to the recognition of specific pathogen toxins by receptor genes in this genotype, resulting in cell death and successful infection through effector triggered susceptibility (ETS). Previous results suggest that ETS-facilitated infection by some host-specific necrotrophs occurs following a gene-for-gene interaction between a host specific toxin and a host receptor protein (reviewed in Oliver and Solomon, 2010). In wheat it has been speculated that the receptor Tsn1 for the host-specific ToxA released by some races of *Pyrenophora tritici-repentis* may be associated with a gene that could be involved in the SA-dependent pathway (Manning et al., 2004). Induction of cell death in the epidermal cells neighboring the infection site (observed in the viability test) along with higher levels of *PR-4* accumulation might have fortified the plant basal defense and increased the level of resistance in 964a-46 compared to Eston.

The putative role of *PR-4* in lentil resistance to *A. lentis* was described previously (Mustafa et al., 2009; Vaghefi et al., 2013). Its antifungal activity was also demonstrated *in vitro* using a recombinant protein (Vaghefi et al., 2013). The suggested involvement of *PR-4* in JA-triggered defense (Thomma et al., 1998) was the reason it was selected for analyzing the role of JA in the present study. *PR-4* expression could explain differences in resistance levels among genotypes. The expression of *PR-4* was not induced in Eston until 24 hpi, yet peaked in CDC Robin at this time. A similar expression peak occurred in 964a-46, but 12 h later and at significantly lower expression levels. Previous studies suggest that the SA signaling pathway is ineffective during the necrotrophic phase of infection and instead JA plays a crucial role (Glazebrook, 2005). The lower susceptibility of 964a-46 to *A. lentis* than that of Eston could be attributed to the potential of 964a-46 to induce higher levels of *PR-4* expression.

Expression of *PR-4* in 964a-46 peaked at 36 hpi, concurrent with the decline in the expression of *PR-1* and *PR-5*, that was expected considering the reciprocal antagonistic effects of SA on JA pathways (Schenk et al., 2000; Kunkel and Brooks, 2002; Glazebrook et al., 2003). Expression of *PR-4* in CDC Robin peaked at 24 hpi, concurrent with low expression of *PR-1* and *PR-5* in this genotype. The antagonistic effects of JA on SA might be the reason for the peak in *PR-1* and *PR-5* expression being absent in CDC Robin at 24 hpi. Suppression of SA-mediated cell death along with the higher levels of *PR-4* expression could be the cause of lower frequency of cell death in CDC Robin and its higher levels of partial resistance than in 964a-46.

The *AOC* expression from 12 to 24 hpi was concomitant with the ranking of disease severity rating of the genotypes, corroborating the putative role of JA signaling in resistance to *A. lentis* as well. Similar to the pattern of *PR-4* expression, *AOC* expression increased at a slower rate in 964a-46 compared to CDC Robin. *AOC* expression peaked about 12 h earlier than *PR-4* (at 24 hpi). As explained previously, *AOC* is a component of the JA biosynthesis pathway (Vick and Zimmerman, 1983). Usually, PR proteins are expressed downstream of the SA and JA signaling cascades, with a time interval between defense activation and expression. This might explain the 12 h delay in the induction of *PR-4* compared to *AOC*.

The current study analyzed the expression of signature genes involved in SA and JA signaling pathways using attached leaf assays. We adopted this method since previous studies on *A. thaliana* suggested distinct reactions in attached and detached leaf assays to hemibiotroph *Colletotrichum* spp. (Liu et al., 2007). The drawback of using attached leaf assays is that it is nearly impossible to completely synchronize the development of fungal individuals on the leaves. We tried to minimize this type of variation by pooling leaflets and including two RNA-extraction batches per biological replicate in the qRT-PCR tests, but independently inoculated time-course experiments are required to confirm observations here.

## Conclusion

Results indicated that genotypes partially resistant to ascochyta blight differ in the timing and magnitude of gene induction associated with the SA and JA signaling pathways. Infection by *A. lentis* caused intensive activation of SA-related genes in 964a-46, suggesting large differences between this and the other resistant genotype CDC Robin and the susceptible check Eston. Expression levels of genes associated with the JA pathway were associated with differences among genotype in the levels of resistance. Microscopy studies suggested that lower disease severity is associated with a lower cell death frequency in CDC Robin. This could not, however, explain the differences between the reaction of 964a-46 and Eston to *A. lentis*. Application of more advanced microscopy with modified staining protocols may enable capture of these differences. The combined results suggested that lentil genotypes carrying different R-genes possess divergent cellular and molecular mechanisms of resistance. Complete understanding of signal transduction pathways activated upon *A. lentis* infection requires further analyses of additional components of SA and JA signaling in relation to the other signals and their downstream pathways.

## Author Contributions

ES conducted experiments under primary supervision of SB and VB. The data were analyzed by SB. Authors equally contributed to the experimental designs. ES drafted the manuscripts with contributions by VB and SB, who also critically reviewed the manuscript. AV was principal investigator and involved in overall design of the project, provided some supervision to ES and critically reviewed the manuscript. All authors have read the manuscript and agreed to its publication.

## Conflict of Interest Statement

The authors declare that the research was conducted in the absence of any commercial or financial relationships that could be construed as a potential conflict of interest.

## Footnotes

1
http://imagej.nih.gov/ij/

2
https://www.ncbi.nlm.nih.gov/tools/primer-blast

## Abbreviations

AOC: allene oxidase cyclase
CLSM: confocal laser scanning microscopy
C_t_: cycle of threshold
hpi: hours post-inoculation
JA: jasmonic acid
PR: pathogenesis-related proteins
qRT-PCR: quantitative real time PCR
R-gene: resistance gene
SA: salicylic acid.
